# Clinical Effect of Traditional Chinese Medicine (An Shen Jiao Tai Yi Zhi Decoction) on Sleep Disorders in Patients With Parkinson’s Disease

**DOI:** 10.62641/aep.v53i3.1809

**Published:** 2025-05-05

**Authors:** Lidan Pu, Xuefei Liu, Chaoyi Fang, Zhiwei Su, Shiyun Sheng, Haolei Yang

**Affiliations:** ^1^Department of Encephalopathy II, Hebei Provincial Hospital of Traditional Chinese Medicine, 050000 Shijiazhuang, Hebei, China; ^2^Traditional Chinese Medicine Diagnosis Teaching and Research Office, Hebei Provincial Hospital of Traditional Chinese Medicine, 050000 Shijiazhuang, Hebei, China; ^3^Department of Encephalopathy I, Hebei Provincial Hospital of Traditional Chinese Medicine, 050000 Shijiazhuang, Hebei, China; ^4^Graduate School, Hebei Provincial Hospital of Traditional Chinese Medicine, 050000 Shijiazhuang, Hebei, China; ^5^Phase I Clinical Trial Research Center of Hebei Provincial Hospital of Traditional Chinese Medicine, 050000 Shijiazhuang, Hebei, China

**Keywords:** traditional Chinese medicine, Parkinson’s disease, sleep disorder, An Shen Jiao Tai Yi Zhi decoction

## Abstract

**Background:**

Current research on An Shen Jiao Tai Yi Zhi decoction remains limited, highlighting the need for further investigation to validate its therapeutic efficacy and elucidate its underlying mechanisms of action. A study was conducted to evaluate the effects of An Shen Jiao Tai Yi Zhi decoction on sleep disorders in patients with Parkinson’s disease.

**Methods:**

The study population comprised 85 patients diagnosed with Parkinson’s disease and sleep disorders at the Department of Encephalopathy of Hebei Provincial Hospital of Traditional Chinese Medicine, between January 2021 and December 2023. In accordance with different treatment methods, they were divided into the Western medicine group (n = 45, conventional Western medicine treatment plus pramipexole hydrochloride) and the traditional Chinese medicine group (n = 40, An Shen Jiao Tai Yi Zhi decoction based on the Western medicine group). To minimize selection bias, propensity score matching (PSM) was employed with a 1:1 ratio, yielding 20 cases in the traditional Chinese medicine group and 20 cases in the Western medicine group. Clinical data, total effective rates, Epworth Sleepiness Scale (ESS) scores, sleep architecture parameters, homocysteine levels, interleukin-1β concentration, and adverse reactions were collected for all participants. Baseline characteristics were balanced between the two groups through PSM. The data were analyzed using *t*-test, chi-square test, and analysis of variance.

**Results:**

PSM matching was performed in a ratio of 1:1, and a total of 40 patients were divided into two groups. No significant differences in clinical characteristics were observed between the groups. The total effective rate of the traditional Chinese medicine group was higher than that of the Western medicine group (*p* < 0.05). Before the intervention, no differences in ESS score, sleep architecture, and related factors were found among the two groups (*p* > 0.05). After 14 days of intervention, the traditional Chinese medicine group exhibited significantly greater improvements across all measured indicators compared to the Western medicine group (*p* < 0.05). Notably, there were no significant differences in the incidence of adverse reactions between the two groups (*p* > 0.05).

**Conclusions:**

An Shen Jiao Tai Yi Zhi decoction demonstrates significant therapeutic efficacy, exhibiting anti-inflammatory properties and promoting changes in sleep architecture, with minimal adverse effects.

## Introduction

Parkinson’s disease (PD) is a prevalent neurodegenerative disorder characterized by a triad of motor symptoms: bradykinesia, muscle rigidity, and resting tremor. This condition impairs motor, cognitive, and neurological functions, potentially increasing the risk of brain lesions. Among the non-motor manifestations, sleep disorder is the most typical, with an estimated incidence of 65–95%. These sleep disorders significantly reduce patients’ quality of life and exacerbate the severity of motor symptoms [1, 2]. Pharmacological interventions for clinical management include dopamine agonists such as pramipexole hydrochloride, as well as levodopa and benserazide. While these agents effectively alleviate motor symptoms, long-term use may lead to adverse effects including diurnal memory loss, excessive daytime somnolence, and cognitive impairment. Furthermore, some patients develop medication dependence and exhibit poor prognosis [3, 4]. In clinical practice, polysomnography (PSG) is the gold standard for objectively assessing sleep quality and structure. Patients with Parkinson’s disease exhibit alterations in sleep structure, characterized by decreased sleep efficiency, difficulty falling asleep, and frequent nocturnal awakenings. PSG studies have revealed that PD patients may experience respiratory disturbances, such as sleep apnea, which directly impair sleep quality. Furthermore, elevated levels of homocysteine (Hcy) and interleukin-1β (IL-1β) have been implicated in the pathogenesis of sleep disorders associated with PD. The elevated levels of Hcy may adversely affect sleep quality in patients with PD, potentially causing damage to the nigrostriatal system through oxidative stress and neurotoxicity [5]. This can aggravate both motor and non-motor symptoms, including sleep disorders. Concurrently, IL-1β plays an important role in inflammatory responses and immune regulation. The increased levels of IL-1β can intensify the inflammatory response, leading to neuronal damage and dysfunction, which in turn may impact sleep structure and quality. Therefore, an appropriate treatment plan should be developed to improve sleep quality while simultaneously addressing the elevated levels of Hcy and IL-1β in PD patients.

In traditional Chinese medicine, sleep disorders associated with Parkinson’s disease are categorized as insomnia, characterized by inadequate sleep duration and depth relative to normal sleep standards. The pathogenesis of sleep disorders in traditional Chinese medicine is attributed to an imbalance between yin and yang, kidney deficiency, reduced essence, and disturbances in mental faculties. Consequently, the therapeutic approach in traditional Chinese medicine aims to reconcile yin and yang, nourish the blood, calm the mind, overcome deficiencies, and reduce excesses [6, 7]. A previous randomized controlled trial [8] demonstrated that traditional Chinese medicine treatments can safely and effectively improve sleep quality and sleep parameters in PD patients. Xiao *et al*. [9] found that traditional Chinese medicine decoction can alleviate symptoms of Parkinson’s disease by improving Hcy levels in patients. Through preclinical animal experiments, Xu *et al*. [10] elucidated the therapeutic potential of traditional Chinese medicine in regulating inflammatory responses. Clinical investigations have corroborated traditional Chinese medicine’s efficacy in improving sleep quality and modulating Hcy and IL-1β levels. In recent years, traditional Chinese medicine formulations have gained increasing interest for their potential applications in treating sleep disorders [11, 12]. An Shen Jiao Tai Yi Zhi decoction is a traditional Chinese herbal formula comprising multiple medicinal components, including cassia twigs, oysters, Salvia miltiorrhiza, and keel. This formulation is conventionally used to address symptoms associated with insomnia and other sleep-related conditions. Each component of the formula is believed to contribute to its therapeutic effects: cassia twigs are hypothesized to regulate yang and dispel wind-related symptoms [13]; oysters are used to calm the mind and settle disturbances; Salvia miltiorrhiza is known for its role in improving blood circulation and cognitive function; and keel is traditionally used to support overall mental health. Collectively, these herbal ingredients are believed to produce a multifaceted effect, including the calming of the mind, reduction of internal wind, and suppression of excessive yang. These actions are postulated to contribute to enhanced sleep quality and cognitive function. Despite its historical use in traditional medicine, contemporary scientific evidence supporting the therapeutic potential of An Shen Jiao Tai Yi Zhi decoction remains limited. This study hypothesizes that An Shen Jiao Tai Yi Zhi decoction may improve sleep disorders in PD patients. The research will involve the administration of the decoction to PD patients experiencing sleep disorders, followed by a comprehensive analysis of its effects, and further investigation of its therapeutic efficacy, to guide subsequent treatments for such patients.

## Materials and Methods

### General Information

A retrospective cohort study was conducted at the Hebei Provincial Hospital of Traditional Chinese Medicine. The study population comprised patients diagnosed with Parkinson’s disease and sleep disorders who were admitted to the Department of Encephalopathy between January 2021 and December 2023. The inclusion criteria were as follows: (1) Parkinson’s disease with Hoehn–Yahr stages Ⅰ–Ⅱ; (2) sleep disorders diagnosed according to the diagnostic criteria of the Clinical Sleep Disorders Manual [14]; and (3) availability of complete clinical data. The exclusion criteria were as follows: (1) severe dementia, mental illness, or cognitive impairment; (2) use of sedatives and hypnotics within a week before the trial; (3) severe heart failure, atrial fibrillation, rheumatic heart disease, pulmonary failure, lung cancer, intracranial space-occupying lesions, or other severe somatic diseases, and inability to cooperate with the examination; (4) suicidal tendency; (5) failure to fully participate in drug treatment.

The minimum effective sample size was calculated according to the comparison formula for two independent sample rates. The significance level and power were established at 5% and 80% (α = 0.05, β = 0.2), respectively.

(1) $$N_{1}=N_{2}=\frac{{\left[Z_{\alpha /2}\,\sqrt{2\,\bar{p}\,\left(1-\bar{p}\right)}+Z_{\beta }\,\sqrt{p_{1}\,\left(1-p_{1}\right)+p_{2}\,\left(1-p_{2}\right)}\right]}^{2}}{{\left(p_{1}-p_{2}\right)}^{2}},$$

where *p* = (*p *_1_ + *p *_2_)/2, and *p *_1_ and *p *_2_ represent the effective rates in the treatment and control groups, respectively. Specifically, the sample sizes of the treatment and control groups were calculated using “Non-Inferiority Tests for the Difference Between Two Proportions” in PASS v15 (NCSS, Kaysville, UT, USA). Based on previous studies [15, 16], the efficacy rates were estimated at 90% for the treatment group and 50% for the control group. Accounting for an anticipated 20% attrition rate due to dropouts and loss-to-follow-up, a minimum sample size of 16 cases per group was calculated, resulting in a total required sample of at least 32 cases. In this study, 85 eligible patients with Parkinson’s disease-associated sleep disorders were identified and divided into the traditional Chinese medicine group (n = 40) and the Western medicine group (n = 45). This study was conducted in accordance with the principles outlined in the Declaration of Helsinki. All participants provided informed consent before enrollment. The study protocol was approved by the institutional ethics committee of Hebei Provincial Hospital of Traditional Chinese Medicine (HBZY2020-KY-162-010).

### Methods

The treatment protocol for the Western medicine group consisted of conventional Western medicine combined with pramipexole hydrochloride (Shi Yao Group Ou Yi Pharmaceutical Company, Hongkong, China, 0.25 mg, National Medical Products Administration (NMPA) approval no.: H20193412). Pramipexole hydrochloride was administered at an initial dose of 0.125 g per administration, with subsequent dose adjustments based on patient response. The maximum daily oral dose was limited to 0.75 g. Dopa and serazide tablets (Shanghai Roche Pharmaceutical Company, Shanghai, China, NMPA approval no.: H10930198, 0.25 g) were administered orally at an initial dose of 0.125 g three times daily. Starting from the second week, the dose was gradually increased until the maintenance dose of 750 mg per day. Dexzopiclone (Chengdu Kanghong Pharmaceutical Group Company, Chengdu, China, NMPA approval no.: H20100074, 3 mg) was initially prescribed at 1 mg before bedtime, and administered once daily. The dose was subsequently increased to 2 or 3 mg based on individual patient requirements and tolerability.

Based on the Western medicine group, the traditional Chinese medicine group received the An Shen Jiao Tai Yi Zhi decoction (10 g of cassia twig, 20 g of keel bone, 20 g of oyster, 15 g of vinegar glans plate, 15 g of Salvia miltiorrhiza, 12 g of *Acorus gramineus* Soland, 10 g of radix curcumae, 20 g of paeoniae alba, 9 g of licorice). The decoction was added to water, and 200 mL of the mixture was administered orally twice daily, in the morning and evening. Both groups underwent a 14-day treatment.

### Observation Indicators

(1) The Parkinson’s Disease Sleep Scale (PDSS) is a scale designed by Chaudhuri *et al*. [17] and specifically used for the evaluation of common sleep problems in patients with Parkinson’s disease. It contains 15 common questions about sleep disorders and measures eight domains: overall quality of sleep at night, sleep onset and maintenance insomnia, nocturnal restlessness, nocturnal psychosis, nocturia, nocturnal motor symptoms, sleep recovery, and daytime medication. Each domain is scored on a scale of 0 to 10, with higher scores indicating greater symptom severity. Treatment efficacy was evaluated after a 14-day intervention using the PDSS reduction rate, calculated as PDSS Reduction Rate = [(Pre-intervention score – Post-intervention score)/Pre-intervention score] × 100%. The efficacy criteria were defined as follows: improved, 89%–10%; ineffective, 0%–9%. Total effective rate = cure rate + improvement rate.

(2) The Epworth Sleepiness Scale (ESS) [18] score of each group was evaluated before the intervention and 14 days after the intervention. This scale was developed by Epworth Hospital and mainly evaluates the degree of daytime sleepiness. It has a total of eight items. Responses are scored on a four-point Likert scale, with total scores interpreted as follows: 0–6 points indicate normal sleepiness, 7–12 mild sleepiness, 13–18 moderate sleepiness, and >18 severe sleepiness. Higher scores correspond to increased levels of daytime sleepiness. The overall Cronbach’s α coefficient of the scale is 0.945 and the α coefficient values of each dimension are greater than 0.85. The split-half reliability coefficient is 0.971. The scale has good reliability and validity and is suitable for the evaluation of hospital patients’ sleepiness.

(3) The sleep architecture was evaluated using PSG (Hong Kong Szymano Medical Supplies Co., Hong Kong, China) [19] before the intervention and 14 days post-intervention. The following parameters were evaluated: sleep efficiency, arousal index, sleep latency, number of awakenings, apnea-hypopnea index (AHI), and minimum oxygen saturation.

(4) Serum levels of related factors were quantified before intervention and 14 days post-intervention. Fasting venous blood samples (3 mL) were collected in the early morning on the day following admission and 14 days after intervention in both groups. Serum was isolated by centrifugation at 3000 r/min for 10 min and stored at –20 °C until analysis. Hcy and IL-1β levels were determined using enzyme-linked immunosorbent assay (ELISA) kits. The Hcy ELISA kit (CSB-E13814h) was obtained from CUSABIO (Wuhan, China), while the IL-1β ELISA kit (EK101BHS) was purchased from Multi Sciences (Hangzhou, China).

(5) Adverse reactions were monitored and quantified across both experimental groups, including constipation (defecation frequency more than three times per week, difficult defecation, dry and firm defecation), anorexia (≥15% weight loss compared to the normal average weight and intermittent gluttony), and nausea (abdominal discomfort and urgency vomiting).

(6) The Hamilton Depression Scale (HAMD) [20] and Hamilton Anxiety Scale (HAMA) [21] scores were determined upon admission in both groups: (i) the HAMD includes 17 items assessing depressive symptoms, with scores serving as reliable indicators of illness severity (<8 points indicate no depression, 8–20 points suggest mild depression, 21–35 points denote moderate depression, and >35 points signify severe depression), (ii) the HAMA consists of 14 items reflecting anxiety symptoms, primarily categorized into somatic and psychogenic anxiety domains (<7 points indicate minimal or no anxiety, 7–21 points suggest mild anxiety, 22–29 points denote moderate anxiety, and >29 points signify severe anxiety).

All scale assessments were conducted by qualified medical professionals within the undergraduate department of the hospital. To ensure standardization and quality control, a comprehensive training program was implemented before the formal study.

### Statistical Analysis

Propensity score matching (PSM) was performed using SPSS v25.0 (IBM, Armonk, NY, USA) to balance the baseline characteristics between the two groups. Covariates for PSM were selected based on baseline variables that exhibited significant differences between the groups. A 1:1 nearest neighbor matching algorithm was employed with a caliper value of 0.02 to balance the sample data. The Shapiro-Wilk test was utilized to assess the normality of data distribution. Continuous variables that followed a normal distribution are expressed as mean ± standard deviation (SD), with comparisons conducted using independent samples *t*-tests. Categorical variables are expressed as frequencies and percentages and analyzed using chi-squared or Fisher’s exact tests. To assess baseline changes and inter-group differences, both absolute differences in scores and percentage changes were calculated. Difference score = pre-treatment value – post-treatment value. Percentage change = [((pre-treatment value – post-treatment value)/pre-treatment value) × 100]. Statistical significance was set at *p* < 0.05 for all analyses.

## Results

### Comparison of Clinical Data of Each Group

The study included a total of 85 patients, with 45 assigned to the Western medicine group and 40 to the Chinese medicine group. Before PSM, statistically significant differences were observed between the groups in age, Hoehn–Yahr stage, body mass index (BMI), HAMD score, and frequency of night awakenings (*p* < 0.05). Following PSM, 20 cases from each group were successfully matched, resulting in balanced baseline characteristics between the groups. No statistically significant differences were observed in matched cohorts (*p* > 0.05) (Table 1).

**Table 1.** **Comparison of clinical data in each group**.

Clinical data		Before matching				After matching			
		Chinese medicine group (n = 40)	Western medicine group (n = 45)	χ^2^/*t*	*p*	Chinese medicine group (n = 20)	Western medicine group (n = 20)	χ^2^/*t*	*p*
Gender									
	Male	23 (57.50)	21 (46.67)	0.995	0.318	9 (45.00)	10 (50.00)	0.100	0.752
	Female	17 (42.50)	24 (53.33)			11 (55.00)	10 (50.00)		
Age (years)		56.38 ± 6.47*	53.42 ± 5.91	2.204	0.030	54.70 ± 6.32	55.05 ± 6.48	0.173	0.864
BMI (kg/m^2^)		21.65 ± 2.34*	23.08 ± 1.76	3.205	0.002	22.55 ± 1.93	22.85 ± 1.60	0.535	0.596
Duration of disease (years)		3.57 ± 1.09	3.82 ± 1.26	0.972	0.334	3.20 ± 0.95	3.40 ± 0.99	0.652	0.518
HAMD (score)		17.94 ± 2.41*	20.11 ± 2.86	3.757	<0.001	18.60 ± 2.06	19.20 ± 2.64	0.801	0.428
HAMA (score)		14.26 ± 1.73	15.03 ± 1.85	1.975	0.052	14.70 ± 1.53	14.95 ± 1.70	0.489	0.628
Degree of education									
	Junior high school and below	17 (42.50)	19 (42.22)	0.008	0.996	9 (45.00)	8 (40.00)	0.483	0.785
	High school	13 (32.50)	15 (33.33)			5 (25.00)	7 (35.00)		
	College degree or above	10 (25.00)	11 (24.44)			6 (30.00)	5 (25.00)		
Marital status									
	Married	18 (45.00)	22 (48.89)	4.607	0.100	10 (50.00)	8 (40.00)	0.622	0.733
	Unmarried	13 (32.50)	20 (44.44)			4 (20.00)	6 (30.00)		
	Divorced or widowed	9 (22.50)	3 (6.67)			6 (30.00)	6 (30.00)		
Hoehn-Yahr stage									
	Class I	28 (70.00)*	22 (48.89)	3.897	0.048	8 (40.00)	11 (55.00)	0.902	0.342
	Grade II	12 (30.00)*	23 (51.11)			12 (60.00)	9 (45.00)		
Time to sleep									
	1–3 h	24 (60.00)	20 (44.44)	2.052	0.152	11 (55.00)	13 (65.00)	0.417	0.519
	>3 h	16 (40.00)	25 (55.56)			9 (45.00)	7 (35.00)		
Number of nighttime awakenings									
	1 to 3 times	22 (55.00)*	14 (31.11)	4.950	0.026	8 (40.00)	6 (30.00)	0.440	0.507
	>3 times	18 (45.00)*	31 (68.89)			12 (60.00)	14 (70.00)		
Nighttime sleep duration									
	4–6 h	16 (40.00)	25 (55.56)	2.052	0.152	10 (50.00)	5 (25.00)	2.667	0.102
	<4 h	24 (60.00)	20 (44.44)			10 (50.00)	15 (75.00)		

Note: Comparison with the Western medicine group, *p < 0.05. BMI, body mass index; HAMD, Hamilton Depression Scale; HAMA, Hamilton Anxiety Scale.

### Comparison of Total Effective Rates in Each Group

The traditional Chinese medicine group exhibited a significantly higher total effective rate (85.00%) compared to the Western medicine group (55.00%, *p* = 0.038) (Table 2).

**Table 2.** **Comparison of the total effective rate of each group**.

Group	Cured	Improved	Ineffective	Total effective rate
Chinese medicine group (n = 20)	7 (35.00)	10 (50.00)	3 (15.00)	17 (85.00)*
Western medicine group (n = 20)	5 (25.00)	6 (30.00)	9 (45.00)	11 (55.00)
χ ^2^				4.286
*p*				0.038

Note: Comparison with the Western medicine group, *p < 0.05.

### Comparison of ESS Scores in Each Group

Before the intervention, no significant difference in ESS scores was observed between the groups (*p* > 0.05). After 14 days of intervention, the traditional Chinese medicine group exhibited significantly lower ESS scores compared to the Western medicine group (*p* < 0.001). Analysis of the difference scores between the two groups revealed significant differences in ESS between the traditional Chinese medicine group and Western medicine group (*p* < 0.001) (Table 3).

**Table 3.** **Comparison of ESS scores and difference scores between groups**.

Group	Western medicine group (n = 20)	Chinese medicine group (n = 20)	*t*	*p*
Before intervention	14.75 ± 2.61	15.35 ± 2.35	–0.764	0.450
After 14 days of intervention	10.10 ± 1.94*	6.70 ± 1.34	6.445	<0.001
Difference scores	–31.33 ± 7.62	–56.03 ± 8.43	9.716	<0.001

Note: Comparison with pre-intervention, *p < 0.05. ESS, Epworth Sleepiness Scale; Difference score = pre-treatment – post-treatment.

### Comparison of Sleep Architecture in Each Group

Before the intervention, no statistically significant differences in sleep architecture were observed between the two groups (*p* > 0.05). After 14 days of intervention, the traditional Chinese medicine group exhibited significantly higher sleep efficiency (*p* < 0.001) and arousal index (*p* = 0.001) compared to the Western medicine group. Conversely, the traditional Chinese medicine group demonstrated significantly lower sleep latency (*p* < 0.001) and AHI (*p* < 0.001) than the Western medicine group. Analysis of percentage changes between groups revealed statistically significant differences in all sleep architecture parameters, except the lowest oxygen saturation (*p* < 0.001) (Table 4).

**Table 4.** **Comparison of sleep architecture and percentage changes in each group**.

Variables		Western medicine group (n = 20)	Chinese medicine group (n = 20)	*t*	*p*
Sleep efficiency (%)	Before intervention	50.75 ± 7.20	51.00 ± 7.55	–0.107	0.915
	After 14 days of intervention	60.10 ± 7.99	69.35 ± 8.02	–3.660	<0.001
	Percentage changes	18.57 ± 4.03	36.69 ± 6.92	–10.120	<0.001
Index of arousal	Before intervention	14.00 ± 2.15	13.75 ± 2.47	0.341	0.735
	After 14 days of intervention	17.60 ± 2.96	21.05 ± 3.22	–3.528	0.001
	Percentage changes	26.17 ± 14.43	56.32 ± 31.94	–3.861	<0.001
Sleep latency (min)	Before intervention	46.80 ± 5.60	47.35 ± 5.81	–0.305	0.762
	After 14 days of intervention	33.45 ± 4.21	26.35 ± 3.12	6.080	<0.001
	Percentage changes	−28.46 ± 4.37	−43.53 ± 9.76	6.300	<0.001
Number of awakenings (times/night)	Before intervention	7.15 ± 2.28	7.05 ± 2.24	0.140	0.889
	After 14 days of intervention	6.35 ± 2.08	5.20 ± 1.70	1.920	0.064
	Percentage changes	−11.07 ± 6.64	−25.74 ± 11.88	4.810	<0.001
AHI (times/h)	Before intervention	36.20 ± 4.72	36.55 ± 4.47	–0.240	0.811
	After 14 days of intervention	30.15 ± 3.51	21.30 ± 3.06	8.500	<0.001
	Percentage changes	−16.43 ± 4.28	−41.73 ± 3.97	19.340	<0.001
Lowest oxygen saturation (%)	Before intervention	94.40 ± 4.10	94.65 ± 4.26	–0.190	0.851
	After 14 days of intervention	96.30 ± 3.51	97.85 ± 2.52	–1.600	0.117
	Percentage changes	2.14 ± 4.76	3.54 ± 4.47	–0.960	0.346

Note: AHI, apnea-hypopnea index; Percentage change = [((pre-treatment value – post-treatment value)/pre-treatment value) × 100].

### Comparison of Related Factors in Each Group

Before the intervention, no statistically significant differences in related factors were observed among the groups (*p* > 0.05). After 14 days of intervention, the traditional Chinese medicine group exhibited significantly lower levels of Hcy and IL-1β compared to the Western medicine group (*p* < 0.001 and *p* = 0.003, respectively). Analysis of the percentage changes between the two groups revealed statistically significant differences in Hcy and IL-1β between the traditional Chinese medicine and Western medicine groups (*p* < 0.001) (Table 5).

**Table 5.** **Comparison of related factors in each group**.

Variables		Western medicine group (n = 20)	Chinese medicine group (n = 20)	*t*	*p*
Hcy (µmol/L)	Before intervention	19.10 ± 2.13	18.55 ± 1.99	0.850	0.403
	After 14 days of intervention	13.05 ± 1.79	9.15 ± 1.31	7.800	<0.001
	Percentage changes	−31.83 ± 3.38	−50.77 ± 4.03	16.070	<0.001
IL-1β (pg/mL)	Before intervention	157.25 ± 20.80	153.45 ± 20.11	0.590	0.560
	After 14 days of intervention	124.35 ± 16.76	109.20 ± 13.34	3.160	0.003
	Percentage changes	−20.86 ± 4.11	−28.68 ± 3.13	6.750	<0.001

Note: Hcy, homocysteine; IL-1β, interleukin-1β; Percentage change = [((pre-treatment value – post-treatment value)/pre-treatment value) × 100].

### Comparison of Adverse Reactions Among Groups

No statistically significant differences were observed in the incidence of adverse reactions between the traditional Chinese medicine group (10.00%) and the Western medicine group (20.00%; *p* > 0.05) (Table 6).

**Table 6.** **Comparison of adverse reactions in each group**.

Group	Constipation	Anorexia	Nausea	Incidence rate
Chinese medicine group (n = 20)	1 (5.00)	1 (5.00)	0 (0.00)	2 (10.00)
Western medicine group (n = 20)	2 (10.00)	1 (5.00)	1 (5.00)	4 (20.00)
χ ^2^				0.196
*p*				0.658

## Discussion

The retrospective analysis of medical records from patients diagnosed with Parkinson’s disease and sleep disorder, who were admitted to the Encephalopathy Department at Hebei Provincial Hospital of Traditional Chinese Medicine, revealed significant therapeutic effects of the An Shen Jiao Tai Yi Zhi decoction. The herbal formulation demonstrated efficacy in attenuating inflammatory responses and modulating sleep architecture in these patients. In addition, the treatment was associated with a low incidence of adverse reactions.

To date, conventional Western medicine remains the most common approach for managing sleep disorders associated with Parkinson’s disease. Dopamine receptor agonists such as pramipexole hydrochloride, levodopa, and benserazide, have demonstrated efficacy in enhancing dopaminergic neurotransmission. These agents effectively augment receptor excitability, confer neuroprotection, prevent oxidative stress damage, improve cerebral blood circulation, and provide trophic support to neurons, collectively contributing to improved sleep quality [22, 23]. Dexzopiclone, a non-benzodiazepine sedative-hypnotic drug, can promote the inhibition of γ-aminobutyric acid receptors in the central nervous system. The mechanism of action results in sedative, hypnotic, and anxiolytic effects. This drug can be absorbed quickly following oral administration. In a randomized controlled trial conducted by Huo S *et al*. [24], dexzopiclone exhibited significant efficacy in improving sleep quality, mitigating sleep disorders, and ameliorating psychiatric symptoms and cognitive function in patients with Alzheimer’s disease and sleep disorders. The study also reported a favorable safety profile for the drug. However, the long-term administration of the drug not only may cause nausea, vomiting, headache, insomnia, drowsiness, and even arrhythmia, hypotension, convulsion, and other adverse reactions but also may cause drug resistance, which is not conducive to the recovery of the disease. Liu M *et al*. [25] showed that the sleep architecture and sleep quality of patients with Parkinson’s disease and sleep disorders considerably improved after treatment with Hua Tan Jie Yu granules, which resolved phlegm, calmed the mind, soothed the liver, and relieved depression. Zhou QH *et al*. [6] concluded that Suanzaoren decoction is an effective treatment for chronic insomnia disorder in adults, with a favorable safety profile. In recent years, traditional Chinese medicine has been used to treat Parkinson’s disease-related sleep disorders, demonstrating not only high efficacy but also advantages such as relatively safe ingredients and ease of administration.

The results showed that the total effective rate of the traditional Chinese medicine group was 85.00%, which was significantly higher than the 55.00% observed in the Western medicine group, suggesting a superior efficacy and safety profile for traditional Chinese medicine. Moreover, no statistically significant difference in adverse reactions was found between the two groups. This may be attributed to the lower toxicity profile of traditional Chinese medicine and its proposed multicomponent, multitarget, and multi-route effects compared with synthetic compounds. In addition, traditional Chinese medicine follows the law of yin and yang of the body and adapts to changes in the nature of yin and yang. The An Shen Jiao Tai Yi Zhi decoction can adapt to the law of yin and yang, aiming to reinforce yang while promoting yin. Notably, as a plant used in traditional Chinese medicine, *Calorus tatarinowii *has been widely used in the treatment of neurodegenerative diseases. Its mechanisms of action include cognitive enhancement, sensory stimulation, alleviation of restlessness, and promotion of mental calmness [26]. Total glucosides of paeony could induce sleep rapidly. Additionally, keel supplementation has been shown to reduce the excitability of the skeletal muscles in patients, potentially contributing to increased sleep duration [27, 28]. The therapeutic approach to sleep disorders generally aims to address the underlying neurophysiological imbalances, yin and yang, calm the mind, and facilitate sleep.

Uysal HA *et al*. [29] observed that patients with Parkinson’s disease often suffer from daytime sleepiness, have difficulty falling asleep, and have reduced sleep maintenance. In a related study, Liu QQ *et al*. [30] investigated the efficacy of traditional Chinese herbal medicine in treating insomnia and reported improvements in sleep quality among affected individuals. These results were similar to those of the present study. Before the intervention, no statistically significant differences were observed in ESS scores or sleep architecture among the three groups. After 14 days of intervention, the ESS score, sleep latency, and AHI in the traditional Chinese medicine group exhibited lower values compared to the Western medicine group. Conversely, sleep efficiency and arousal index were higher in the traditional Chinese medicine group. This discrepancy can be attributed to the utilization of PSG as the main diagnostic tool for measuring sleep. PSG enables comprehensive analysis of sleep architecture and processes, providing a detailed characterization of insomnia severity. Patients with Parkinson’s disease typically experience diminished sleep quality due to a combination of factors. These include motor symptoms such as tremors, and muscle stiffness, as well as potential excitatory effects of certain drugs on the cerebral cortex. The sleep structure of patients with Parkinson’s disease exhibits notable alterations that impact overall sleep quality. Specifically, these patients demonstrate reduced sleep efficiency, and increased sleep fragmentation characterized by frequent nocturnal awakenings, and prolonged sleep latency. Additionally, respiratory disturbances have been observed, resulting in a decline in the minimum oxygen saturation or even apnea [31, 32]. To elucidate the effects of the intervention on sleep architecture, this study employed PSG to analyze the sleep architecture of PD patients both pre- and post-intervention. The efficacy of An Shen Jiao Tai Yi Zhi decoction in alleviating sleep disorders was demonstrated by significant improvements in traditional Chinese medicine indices following a 14-day intervention. The decoction’s therapeutic effects were comparable to those of conventional pharmaceutical treatments. Specifically, baipeony has been shown to induce sleep quickly, while keel can prolong total sleep duration and reduce nocturnal activity [33]. Oysters can enhance yang, strengthen yin, and promote mental calmness. In addition, studies have shown that the An Shen Jiao Tai Yi Zhi decoction can increase norepinephrine and dopamine levels in the thalamus and cerebral cortex and upregulate the expression of 5-hydroxytryptamine and 5-hydroxyindoleacetic acid. These neurochemical changes are associated with the regulation of brain neurotransmitters, leading to improvements in sleep quality among patients [34].

In this study, after a 14-day intervention, the traditional Chinese medicine group exhibited significantly lower levels of Hcy and IL-1β compared to the Western medicine group. Hcy, a sulfur-containing amino acid secreted during methionine metabolism, is typically expressed at low levels but shows elevated concentrations upon the onset of a neurodegenerative disease. Elevated Hcy levels have been closely related to sleep disorders in Parkinson’s disease patients, making it an important indicator for assessing sleep disorders in those patients [35, 36]. IL-1β, a member of the chemokine family of cytokines, is a common inflammatory mediator in the nervous system. This cytokine plays a synergistic role along with antigens, stimulating T cells, and promoting B cell proliferation and differentiation, ultimately leading to inflammatory responses [37, 38].

This study provides insights into potential approaches for addressing sleep quality in patients with Parkinson’s disease. However, this study has some limitations. First, the study was constrained by a small sample size, comprising only patients diagnosed with Parkinson’s disease who were admitted to the hospital. These samples may not be sufficiently representative to adequately reflect the overall therapeutic efficacy of Chinese herbal medicine for sleep disorders in these patients. Secondly, the short-term nature of the clinical trials may not fully capture the long-term efficacy of traditional Chinese medicines.

Parkinson’s disease is a chronic, progressive neurodegenerative disease characterized by a long and complex clinical course. Sleep disturbances associated with this condition may persist and change over time. The lack of longitudinal follow-up data presents a significant limitation in comprehensively assessing the long-term therapeutic efficacy of traditional Chinese medicine, making it difficult to determine the actual impact of this medicine on improving patients’ quality of life over a long period. Finally, this study did not consider other potential influencing factors such as patients’ lifestyle, comorbidities, and concurrent medication, which may have introduced systematic bias into the results. To overcome these limitations, future studies with larger sample sizes are needed to improve the reliability and generalizability of the findings. At the same time, long-term follow-up protocols should be implemented to regularly assess patients and obtain more data on long-term therapeutic effects. This will not only help understand the durability of traditional Chinese medicine treatments but also validate their therapeutic efficacy for sleep disorders associated with Parkinson’s disease. Through these improvements, we can more comprehensively evaluate the role of traditional Chinese medicine in treating sleep disorders in patients with Parkinson’s disease and provide a more solid scientific basis for clinical practice.

## Conclusions

The Shen Jiao Tai Yi Zhi decoction demonstrated a therapeutic effect in patients with Parkinson’s disease, significantly improving sleep quality, reducing inflammatory responses, alleviating sleep disorders, and modulating sleep architecture. In addition, this formulation exhibited a favorable safety profile with minimal adverse effects.

## Availability of Data and Materials

Data to support the findings of this study are available on reasonable request from the corresponding author.
